# *N*-Cyclo­heptyl­idene-*N*′-(2,4-dinitro­phenyl)hydrazine

**DOI:** 10.1107/S1600536809002657

**Published:** 2009-01-23

**Authors:** Reza Kia, Hoong-Kun Fun, Hadi Kargar

**Affiliations:** a X-ray Crystallography Unit, School of Physics, Universiti Sains Malaysia, 11800 USM, Penang, Malaysia; b Department of Chemistry, School of Science, Payame Noor University (PNU), Ardakan, Yazd, Iran

## Abstract

The title compound, C_13_H_16_N_4_O_4_, is a new hydrazone. An intra­molecular N—H⋯O hydrogen bond generates a six-membered ring, producing an *S*(6) ring motif. The nitro groups in the *ortho* and *para* positions are almost coplanar with the benzene ring to which they are bound, making dihedral angles of 0.60 (11) and 3.18 (11)°, respectively. Pairs of inter­molecular C—H⋯O hydrogen bonds link neighbouring mol­ecules into inversion dimers with *R*_2_^2^(10) motifs. The crystal structure is further stabilized by inter­molecular π–π inter­actions, with a benzene centroid-to-centroid distance of 3.6601 (4) Å.

## Related literature

For details of hydrogen-bond motifs, see: Bernstein *et al.* (1995 ▶). For related literature on the applications of hydrazone, see, for example: Niknam *et al.*, (2005 ▶); Guillaumont & Nakamura (2000 ▶); Raj & Kurup (2006 ▶); Okabe *et al.* (1993 ▶).
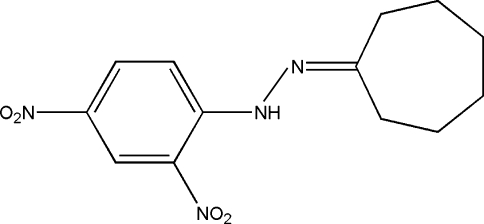


## Experimental

### 

#### Crystal data

C_13_H_16_N_4_O_4_
*M* *_r_* = 292.30
Monoclinic, 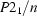

*a* = 6.9721 (1) Å
*b* = 23.7359 (5) Å
*c* = 8.2274 (2) Å
β = 102.351 (1)°
*V* = 1330.03 (5) Å^3^
*Z* = 4
Mo *K*α radiation
μ = 0.11 mm^−1^
*T* = 100.0 (1) K
0.51 × 0.45 × 0.08 mm

#### Data collection

Bruker SMART APEXII CCD area-detector diffractometer
Absorption correction: multi-scan (*SADABS*; Bruker, 2005 ▶) *T*_min_ = 0.946, *T*_max_ = 0.991
26146 measured reflections
5824 independent reflections
4916 reflections with *I* > 2σ(*I*)
*R*_int_ = 0.026

#### Refinement

*R*[*F* ^2^ > 2σ(*F* ^2^)] = 0.039
*wR*(*F* ^2^) = 0.113
*S* = 1.04
5824 reflections
194 parameters
H atoms treated by a mixture of independent and constrained refinement
Δρ_max_ = 0.43 e Å^−3^
Δρ_min_ = −0.30 e Å^−3^

### 

Data collection: *APEX2* (Bruker, 2005 ▶); cell refinement: *SAINT* (Bruker, 2005 ▶); data reduction: *SAINT*; program(s) used to solve structure: *SHELXTL* (Sheldrick, 2008 ▶); program(s) used to refine structure: *SHELXTL*; molecular graphics: *SHELXTL*; software used to prepare material for publication: *SHELXTL* and *PLATON* (Spek, 2003 ▶).

## Supplementary Material

Crystal structure: contains datablocks global, I. DOI: 10.1107/S1600536809002657/kj2114sup1.cif

Structure factors: contains datablocks I. DOI: 10.1107/S1600536809002657/kj2114Isup2.hkl

Additional supplementary materials:  crystallographic information; 3D view; checkCIF report

## Figures and Tables

**Table 1:** Hydrogen-bond geometry (Å, °)

*D*—H⋯*A*	*D*—H	H⋯*A*	*D*⋯*A*	*D*—H⋯*A*
N1—H1N1⋯O2	0.888 (14)	1.947 (14)	2.6225 (9)	131.7 (12)
C2—H2*A*⋯O3^i^	0.95	2.52	3.3165 (10)	142

Symmetry code: (i) 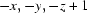.

**Fig. 1.**
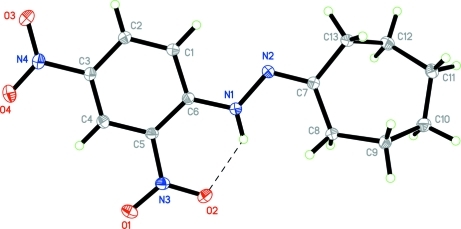
View of the molecular structure of the title compound with atom labels and 50% probability ellipsoids for non-H atoms. Intramolecular hydrogen bond is shown as dash lines.

**Fig. 2.**
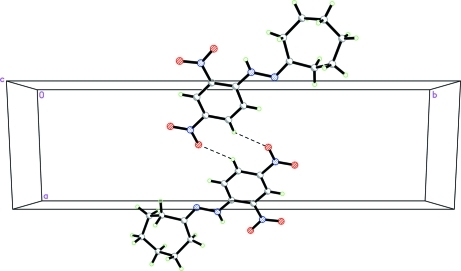
The crystal packing of the title compound, viewed down the c-axis showing dimer formation. Intermolecular hydrogen bonds are shown as dashed lines.

## supplementary crystallographic information

### Comment

2,4-Dinitrophenylhydrazones play an important role as stabilizers for the detection and protection of the carbonyl group (Niknam *et al.*, 2005). 2,4-Dinitrophenylhydrazone derivatives are widely used in as dyes (Guillaumont & Nakamura, 2000). They are also found to have versatile coordinating abilities towards different metal ions (Raj & Kurup, 2006). In addition, some phenylhydrazone derivatives have been shown to be potentially DNA-damaging and mutagenic agents (Okabe *et al.*, 1993).

The title compound (Fig. 1) is a new hydrazone. An intramolecular N—H···O hydrogen bond generates a six-membered ring, producing an *S(6)* ring motif (Bernstein *et al.*, 1995). The nitro groups in the *ortho* and *para* positions are almost coplanar with the benzene ring to which they are bound, making dihedral angles of 0.60 (11)° and 3.18 (11)°, respectively. The cycloheptanone ring is puckered with a total puckering amplitude, Q = 0.7820 (8) Å. Pairs of intermolecular C—H···O hydrogen bonds link neighbouring molecules into dimers with *R_2_^2^(10)* motifs (Table 1, Fig. 2). The crystal structure is further stabilized by intermolecular π–π interactions [*Cg*1···*Cg*1(1 - *x*, -*y*, 1 - *z*) = 3.6601 (4) Å, with *Cg* the centroid of the benzene ring].

### Experimental

The title compound was synthesized based on the reported procedure (Okabe *et al.* 1993). Single crystals suitable for X-ray diffraction analysis were grown by slow evaporation of a saturated solution of the resulted compound in DMF.

### Refinement

The H atom bound to N1 was located from the difference Fourier map and refined freely, see Table 1. The rest of the H atoms were positioned geometrically and refined in a riding model approximation with C—H = 0.95–0.99 Å and *U*_iso_(H) = 1.2*U*_eq_(C).

### Crystal data

**Table:**

C_13_H_16_N_4_O_4_	*F*(000) = 616
*M**_r_* = 292.30	*D*_x_ = 1.460 Mg m^−^^3^
Monoclinic, *P*2_1_/*n*	Mo *K*α radiation, λ = 0.71073 Å
Hall symbol: -P 2yn	Cell parameters from 9967 reflections
*a* = 6.9721 (1) Å	θ = 2.7–40.2°
*b* = 23.7359 (5) Å	µ = 0.11 mm^−^^1^
*c* = 8.2274 (2) Å	*T* = 100 K
β = 102.351 (1)°	Plate, yellow
*V* = 1330.03 (5) Å^3^	0.51 × 0.45 × 0.08 mm
*Z* = 4	

### Data collection

**Table:**

Bruker SMART APEXII CCD area-detector diffractometer	5824 independent reflections
Radiation source: fine-focus sealed tube	4916 reflections with *I* > 2σ(*I*)
graphite	*R*_int_ = 0.026
φ and ω scans	θ_max_ = 35.0°, θ_min_ = 1.7°
Absorption correction: multi-scan (*SADABS*; Bruker, 2005)	*h* = −11→11
*T*_min_ = 0.946, *T*_max_ = 0.991	*k* = −38→38
26146 measured reflections	*l* = −12→13

### Refinement

**Table:**

Refinement on *F*^2^	Primary atom site location: structure-invariant direct methods
Least-squares matrix: full	Secondary atom site location: difference Fourier map
*R*[*F*^2^ > 2σ(*F*^2^)] = 0.039	Hydrogen site location: inferred from neighbouring sites
*wR*(*F*^2^) = 0.113	H atoms treated by a mixture of independent and constrained refinement
*S* = 1.04	*w* = 1/[σ^2^(*F*_o_^2^) + (0.0637*P*)^2^ + 0.2167*P*] where *P* = (*F*_o_^2^ + 2*F*_c_^2^)/3
5824 reflections	(Δ/σ)_max_ < 0.001
194 parameters	Δρ_max_ = 0.43 e Å^−^^3^
0 restraints	Δρ_min_ = −0.30 e Å^−^^3^

### Special details

**Table:**

Experimental. The low-temperature data was collected with the Oxford Cryosystem Cobra low-temperature attachment.
Geometry. All e.s.d.'s (except the e.s.d. in the dihedral angle between two l.s. planes) are estimated using the full covariance matrix. The cell e.s.d.'s are taken into account individually in the estimation of e.s.d.'s in distances, angles and torsion angles; correlations between e.s.d.'s in cell parameters are only used when they are defined by crystal symmetry. An approximate (isotropic) treatment of cell e.s.d.'s is used for estimating e.s.d.'s involving l.s. planes.
Refinement. Refinement of *F*^2^ against ALL reflections. The weighted *R*-factor *wR* and goodness of fit *S* are based on *F*^2^, conventional *R*-factors *R* are based on *F*, with *F* set to zero for negative *F*^2^. The threshold expression of *F*^2^ > σ(*F*^2^) is used only for calculating *R*-factors(gt) *etc*. and is not relevant to the choice of reflections for refinement. *R*-factors based on *F*^2^ are statistically about twice as large as those based on *F*, and *R*- factors based on ALL data will be even larger.

### Fractional atomic coordinates and isotropic or equivalent isotropic displacement parameters (Å^2^)

**Table:**

	*x*	*y*	*z*	*U*_iso_*/*U*_eq_	
O1	0.71910 (9)	−0.13041 (2)	0.17783 (8)	0.02285 (12)	
O2	0.81378 (8)	−0.04726 (2)	0.11828 (8)	0.02030 (12)	
O3	0.01157 (8)	−0.08982 (3)	0.49098 (7)	0.02059 (12)	
O4	0.17434 (9)	−0.16037 (2)	0.41905 (8)	0.02340 (13)	
N1	0.62623 (9)	0.04321 (3)	0.18111 (8)	0.01434 (11)	
N2	0.58494 (9)	0.10007 (2)	0.19219 (8)	0.01495 (11)	
N3	0.70085 (9)	−0.07900 (3)	0.17430 (8)	0.01533 (11)	
N4	0.14350 (9)	−0.10960 (3)	0.42854 (8)	0.01614 (12)	
C1	0.34898 (10)	0.02454 (3)	0.30373 (9)	0.01400 (12)	
H1A	0.3228	0.0638	0.3054	0.017*	
C2	0.23044 (10)	−0.01240 (3)	0.36477 (9)	0.01428 (12)	
H2A	0.1231	0.0012	0.4077	0.017*	
C3	0.26847 (10)	−0.07040 (3)	0.36355 (8)	0.01365 (12)	
C4	0.42249 (10)	−0.09135 (3)	0.30138 (8)	0.01385 (12)	
H4A	0.4470	−0.1307	0.3017	0.017*	
C5	0.54189 (9)	−0.05386 (3)	0.23798 (8)	0.01284 (11)	
C6	0.51047 (9)	0.00541 (3)	0.23793 (8)	0.01249 (11)	
C7	0.71332 (10)	0.13537 (3)	0.16026 (9)	0.01369 (12)	
C8	0.90306 (10)	0.11873 (3)	0.11327 (9)	0.01459 (12)	
H8A	0.8734	0.1078	−0.0056	0.018*	
H8B	0.9549	0.0849	0.1784	0.018*	
C9	1.06529 (10)	0.16349 (3)	0.13962 (9)	0.01612 (13)	
H9A	1.0778	0.1796	0.2524	0.019*	
H9B	1.1911	0.1447	0.1362	0.019*	
C10	1.03488 (12)	0.21200 (3)	0.01374 (11)	0.02085 (15)	
H10A	1.1629	0.2307	0.0187	0.025*	
H10B	0.9920	0.1959	−0.0993	0.025*	
C11	0.88606 (11)	0.25661 (3)	0.03869 (10)	0.01808 (13)	
H11A	0.9336	0.2747	0.1483	0.022*	
H11B	0.8801	0.2860	−0.0475	0.022*	
C12	0.67896 (11)	0.23445 (3)	0.03073 (10)	0.01820 (14)	
H12A	0.6359	0.2131	−0.0741	0.022*	
H12B	0.5890	0.2669	0.0276	0.022*	
C13	0.66109 (11)	0.19625 (3)	0.17764 (10)	0.01816 (13)	
H13A	0.7471	0.2115	0.2797	0.022*	
H13B	0.5242	0.1982	0.1930	0.022*	
H1N1	0.727 (2)	0.0296 (6)	0.1432 (17)	0.036 (3)*	

### Atomic displacement parameters (Å^2^)

**Table:**

	*U*^11^	*U*^22^	*U*^33^	*U*^12^	*U*^13^	*U*^23^
O1	0.0256 (3)	0.0139 (2)	0.0313 (3)	0.0047 (2)	0.0112 (2)	−0.0015 (2)
O2	0.0173 (2)	0.0201 (2)	0.0264 (3)	−0.00068 (19)	0.0112 (2)	−0.0007 (2)
O3	0.0189 (2)	0.0239 (3)	0.0216 (3)	−0.0013 (2)	0.0100 (2)	0.0012 (2)
O4	0.0262 (3)	0.0139 (2)	0.0317 (3)	−0.0030 (2)	0.0096 (2)	0.0025 (2)
N1	0.0135 (2)	0.0119 (2)	0.0186 (3)	−0.00005 (18)	0.00577 (19)	0.00008 (19)
N2	0.0142 (2)	0.0117 (2)	0.0198 (3)	0.00064 (18)	0.0055 (2)	0.0012 (2)
N3	0.0145 (2)	0.0153 (3)	0.0166 (3)	0.00157 (19)	0.00412 (19)	−0.0017 (2)
N4	0.0161 (3)	0.0163 (3)	0.0162 (3)	−0.0022 (2)	0.0037 (2)	0.0015 (2)
C1	0.0132 (3)	0.0127 (3)	0.0166 (3)	0.0010 (2)	0.0045 (2)	0.0005 (2)
C2	0.0133 (3)	0.0144 (3)	0.0158 (3)	0.0007 (2)	0.0045 (2)	0.0008 (2)
C3	0.0139 (3)	0.0132 (3)	0.0141 (3)	−0.0011 (2)	0.0036 (2)	0.0009 (2)
C4	0.0145 (3)	0.0128 (3)	0.0140 (3)	−0.0004 (2)	0.0025 (2)	−0.0003 (2)
C5	0.0122 (3)	0.0128 (3)	0.0138 (3)	0.0008 (2)	0.0036 (2)	−0.0014 (2)
C6	0.0119 (3)	0.0127 (3)	0.0127 (3)	−0.00011 (19)	0.00235 (19)	0.0000 (2)
C7	0.0131 (3)	0.0125 (3)	0.0160 (3)	0.0006 (2)	0.0043 (2)	0.0009 (2)
C8	0.0133 (3)	0.0130 (3)	0.0184 (3)	0.0002 (2)	0.0054 (2)	−0.0004 (2)
C9	0.0125 (3)	0.0149 (3)	0.0210 (3)	−0.0004 (2)	0.0037 (2)	0.0026 (2)
C10	0.0200 (3)	0.0173 (3)	0.0284 (4)	0.0029 (2)	0.0121 (3)	0.0069 (3)
C11	0.0199 (3)	0.0137 (3)	0.0218 (3)	0.0009 (2)	0.0071 (2)	0.0032 (2)
C12	0.0170 (3)	0.0142 (3)	0.0233 (3)	0.0029 (2)	0.0040 (2)	0.0029 (2)
C13	0.0191 (3)	0.0128 (3)	0.0256 (3)	0.0016 (2)	0.0115 (3)	0.0003 (2)

### Geometric parameters (Å, °)

**Table:**

O1—N3	1.2265 (8)	C7—C13	1.5044 (10)
O2—N3	1.2471 (8)	C7—C8	1.5080 (10)
O3—N4	1.2380 (9)	C8—C9	1.5330 (10)
O4—N4	1.2296 (8)	C8—H8A	0.9900
N1—C6	1.3551 (9)	C8—H8B	0.9900
N1—N2	1.3871 (8)	C9—C10	1.5328 (10)
N1—H1N1	0.887 (14)	C9—H9A	0.9900
N2—C7	1.2934 (9)	C9—H9B	0.9900
N3—C5	1.4517 (9)	C10—C11	1.5269 (11)
N4—C3	1.4525 (9)	C10—H10A	0.9900
C1—C2	1.3717 (10)	C10—H10B	0.9900
C1—C6	1.4241 (10)	C11—C12	1.5249 (11)
C1—H1A	0.9500	C11—H11A	0.9900
C2—C3	1.4026 (10)	C11—H11B	0.9900
C2—H2A	0.9500	C12—C13	1.5373 (11)
C3—C4	1.3772 (10)	C12—H12A	0.9900
C4—C5	1.3938 (10)	C12—H12B	0.9900
C4—H4A	0.9500	C13—H13A	0.9900
C5—C6	1.4237 (9)	C13—H13B	0.9900
			
C6—N1—N2	118.31 (6)	C7—C8—H8B	108.2
C6—N1—H1N1	117.1 (9)	C9—C8—H8B	108.2
N2—N1—H1N1	124.6 (9)	H8A—C8—H8B	107.4
C7—N2—N1	117.04 (6)	C10—C9—C8	115.71 (6)
O1—N3—O2	122.64 (6)	C10—C9—H9A	108.4
O1—N3—C5	118.93 (6)	C8—C9—H9A	108.4
O2—N3—C5	118.43 (6)	C10—C9—H9B	108.4
O4—N4—O3	123.61 (7)	C8—C9—H9B	108.4
O4—N4—C3	118.53 (6)	H9A—C9—H9B	107.4
O3—N4—C3	117.86 (6)	C11—C10—C9	115.46 (6)
C2—C1—C6	121.51 (6)	C11—C10—H10A	108.4
C2—C1—H1A	119.2	C9—C10—H10A	108.4
C6—C1—H1A	119.2	C11—C10—H10B	108.4
C1—C2—C3	119.69 (6)	C9—C10—H10B	108.4
C1—C2—H2A	120.2	H10A—C10—H10B	107.5
C3—C2—H2A	120.2	C12—C11—C10	114.80 (6)
C4—C3—C2	121.37 (6)	C12—C11—H11A	108.6
C4—C3—N4	118.83 (6)	C10—C11—H11A	108.6
C2—C3—N4	119.79 (6)	C12—C11—H11B	108.6
C3—C4—C5	118.95 (6)	C10—C11—H11B	108.6
C3—C4—H4A	120.5	H11A—C11—H11B	107.5
C5—C4—H4A	120.5	C11—C12—C13	113.89 (6)
C4—C5—C6	121.79 (6)	C11—C12—H12A	108.8
C4—C5—N3	115.84 (6)	C13—C12—H12A	108.8
C6—C5—N3	122.37 (6)	C11—C12—H12B	108.8
N1—C6—C5	123.46 (6)	C13—C12—H12B	108.8
N1—C6—C1	119.84 (6)	H12A—C12—H12B	107.7
C5—C6—C1	116.69 (6)	C7—C13—C12	115.46 (6)
N2—C7—C13	114.26 (6)	C7—C13—H13A	108.4
N2—C7—C8	124.44 (6)	C12—C13—H13A	108.4
C13—C7—C8	121.29 (6)	C7—C13—H13B	108.4
C7—C8—C9	116.32 (6)	C12—C13—H13B	108.4
C7—C8—H8A	108.2	H13A—C13—H13B	107.5
C9—C8—H8A	108.2		
			
C6—N1—N2—C7	170.12 (6)	C4—C5—C6—N1	178.35 (6)
C6—C1—C2—C3	0.28 (10)	N3—C5—C6—N1	−0.87 (10)
C1—C2—C3—C4	−0.32 (10)	C4—C5—C6—C1	−0.89 (9)
C1—C2—C3—N4	179.83 (6)	N3—C5—C6—C1	179.89 (6)
O4—N4—C3—C4	−3.17 (10)	C2—C1—C6—N1	−178.97 (6)
O3—N4—C3—C4	176.68 (6)	C2—C1—C6—C5	0.30 (10)
O4—N4—C3—C2	176.69 (7)	N1—N2—C7—C13	−178.94 (6)
O3—N4—C3—C2	−3.46 (10)	N1—N2—C7—C8	−0.34 (10)
C2—C3—C4—C5	−0.26 (10)	N2—C7—C8—C9	−159.23 (7)
N4—C3—C4—C5	179.60 (6)	C13—C7—C8—C9	19.27 (10)
C3—C4—C5—C6	0.88 (10)	C7—C8—C9—C10	−74.34 (8)
C3—C4—C5—N3	−179.85 (6)	C8—C9—C10—C11	76.57 (9)
O1—N3—C5—C4	0.89 (9)	C9—C10—C11—C12	−59.37 (9)
O2—N3—C5—C4	−179.00 (6)	C10—C11—C12—C13	68.70 (9)
O1—N3—C5—C6	−179.84 (6)	N2—C7—C13—C12	−131.11 (7)
O2—N3—C5—C6	0.26 (10)	C8—C7—C13—C12	50.25 (9)
N2—N1—C6—C5	−177.98 (6)	C11—C12—C13—C7	−84.10 (8)
N2—N1—C6—C1	1.24 (10)		

### Hydrogen-bond geometry (Å, °)

**Table:**

*D*—H···*A*	*D*—H	H···*A*	*D*···*A*	*D*—H···*A*
N1—H1N1···O2	0.888 (14)	1.947 (14)	2.6225 (9)	131.7 (12)
C2—H2A···O3^i^	0.95	2.52	3.3165 (10)	142

Symmetry codes: (i) −*x*, −*y*, −*z*+1.
